# Influence of Cochlear Anatomy on Intraoperative Electrically Evoked Compound Action Potentials

**DOI:** 10.3390/jcm13164716

**Published:** 2024-08-12

**Authors:** Nawaf Fatani, Yassin Abdelsamad, Abdulrahman Alsanosi

**Affiliations:** 1 King Abdullah Ear Specialist Center (KAESC), College of Medicine, King Saud University Medical City (KSUMC), King Saud University, Riyadh 11411, Saudi Arabia; 2 Research Department, MED-EL GmbH, Riyadh 12631, Saudi Arabia

**Keywords:** cochlear implant, cochlear implantation, evoked compound action potential, cochlear duct length

## Abstract

**Objective:** The electrically evoked compound action potential (ECAP) is an objective measure to indirectly assess spiral ganglion neurons. The ECAP provides inputs about the prognoses of cochlear implant (CI) recipients. Several factors such as cochlear morphology can affect ECAP measurements. This study aims to investigate the variation effect of cochlear parameters on intraoperative ECAP thresholds. **Methods:** This is a retrospective study on patients who underwent CI surgery with normal inner ear morphology at our center between 2017 and 2023. Cochlear anatomical parameters, including diameter (A value), width (B value), and height (H value), as well as cochlear duct length (CDL), were measured pre-operatively using OTOPLAN software (Version 3.0). Cochlear implant intraoperative objective measures were also collected. The correlation between the cochlear parameters and intraoperative objective measures was studied. **Results:** A total of 45 patients underwent cochlear implantation. The mean age was 2.4 ± 0.9 years. The mean CDL and cochlear coverage values were 33.2 ± 2.0 mm and 76.0 ± 5.7%, respectively. The ECAP threshold increased toward basal electrodes, with ECAP values as follows: apical 13.1 ± 3.8; middle 14.3 ± 3.7; and basal 15.6 ± 4.8. Additionally, the A, B, and H values showed a positive correlation with ECAP thresholds in different cochlear regions. The B value showed a significant moderate correlation with ECAP thresholds in the middle and basal electrodes but not in the apical electrodes. **Conclusions:** Cochlear anatomical parameters correlate with intraoperative ECAP thresholds. The B value showed a significant association with ECAP thresholds in the middle and basal electrodes. These findings could delineate the impact of the B value in CI and optimize electrode selection. Further research is required to study this correlation and its impact on postoperative outcomes.

## 1. Introduction 

Cochlear anatomy in humans is known to have variations in morphology and size [1,2,3], and about 25% of congenital sensorineural hearing loss is associated with bony malformation of the inner ear that is identified using computed tomography (CT). Although the cochlea reaches adult size by 16 to 20 weeks of gestation [4], studies have shown that the organ of Corti can vary in length among individuals with normal appearance of the cochlea; moreover, the number of cochlear turns and otic capsule size can vary [5]. A study by Erixon et al. reported that each cochlea has its own “fingerprint” due to variability in design and characteristics.

Cochlear implantation (CI) is a well-known treatment for patients who are diagnosed with severe to profound sensorineural hearing loss without any benefit from using regular hearing aids. The spiral ganglion receives electrical stimulation through intracochlear electrodes, which is then transmitted to the auditory nerve. However, CI surgery can cause significant trauma that leads to neural degeneration during electrode insertion [6,7]. Furthermore, patients who underwent CI surgery with residual hearing showed improved speech outcomes [7]. Therefore, understanding cochlear variations in anatomy and dimension is a significant development in minimizing the trauma of electrode insertion during CI surgery.

Recently, more strategies toward minimizing the traumatic insertion of cochlear implant electrode arrays and preservation of residual hearing have been developed [8]. Moreover, the position of the electrode can affect the performance of CI patients. Thus, the anatomy of the cochlea, electrode design, and surgical technique can influence the electrode insertion and its final position [7]. In addition, a wide range of frequencies should be stimulated by the electrode array; to achieve this, a variety of electrode designs and lengths are available. Measurement of the cochlear duct length (CDL) is performed to select the appropriate electrode and minimize intracochlear trauma [6].

The electrically evoked compound action potential (ECAP) is considered a viable objective measure that is determined by recording the electrical stimulation of the electrode array to assess the functionality of spiral ganglion neurons. The ECAP can provide inputs about the prognosis of the cochlear implant recipient, measuring the positive influence of a large number of stimulated neurons on the outcome for CI patients. A study showed that the ECAP amplitude growth function (AGF) decreases from apical to basal electrodes, suggesting that there are more and/or healthier neurons in the apical region [9]. Moreover, the correlation of the ECAP AGF to different patients’ parameters was studied, showing a negative correlation to the age at implantation and duration of deafness. However, no correlation was found between the ECAP and the etiology of hearing loss and the length of the electrode. In addition, increased cochlear coverage leads to better speech perception because more SGN is stimulated by greater cochlear coverage. As a result, the cochlear coverage and ECAP slope may be correlated and should be investigated further [9]. Moreover, anatomical variation in the cochlea can influence electrode position and cochlear coverage [10].

Furthermore, Mlynski et al. reported a study of cochlear duct length in association with the ECAP in CI patients. The study concluded that there is a correlation between the ECAP slopes and patient anatomy and reported that cochlear duct length measurement is a reliable method to determine electrode position in the cochlea, thus indicating the potential to improve patient care by combining the ECAP and anatomical parameters [11]. Another study assessed the CI electrode position using imaging in relation to different objective measures and impedances. The ECAP showed a consistent pattern; however, the variability was high between patients and the position of the CI electrode in the cochlea. High neural excitation evident in the ECAP recordings was observed when the electrode was closer to the apical area and modiolus. Many factors can affect the ECAP response in CI patients, and this should be explored for further understanding of this variability [10].

Studies have reported variation in CDL based on gender, head size, and ethnicity, as well as geographical area [6,12,13,14,15]. However, the effect of this anatomical variation on the intraoperative objective measures is still not extensively reported in the literature. Therefore, this study aims to investigate the variation in cochlear parameters, including diameter (A value), width (B value), and height (H value), as well as cochlear duct length (CDL) and its effect on intraoperative ECAP thresholds during cochlear implant surgery and impedance values. 

## 2. Methods and Materials 

### 2.1. Study Design and Population

This is a retrospective study on patients who underwent CI at our tertiary center between 2017 and 2023. This study included pediatric patients diagnosed with profound sensorineural hearing loss who underwent unilateral or bilateral CI surgery. The included patients were also required to have normal inner and middle ear and cochleovestibular nerve morphology, which was determined using high-resolution computer tomography (HRCT) and magnetic resonance imaging (MRI). Cochlear duct length was measured pre-operatively using OTOPLAN software (Version 3.0). Only patients who received MED-El implants were included. We excluded patients who had surgical complications, incomplete electrode array insertion, and insufficient data. CI intraoperative objective measures were collected. We studied the correlation between different cochlear parameters and the intraoperative objective measures. The study protocol was reviewed and approved by the institutional review board (Reference No. 23/0558/IRB).

### 2.2. Cochlear Duct Length Measurements

**Figure 1:**
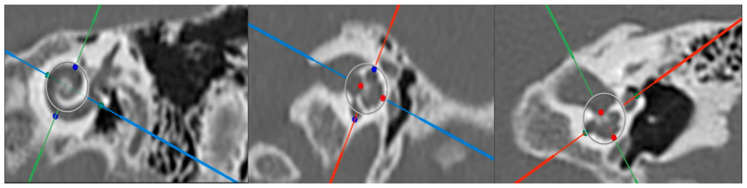
Cochlear parameters using OTOPLAN software, showing A value (green dots), B value (blue dots), and H value (red dots).

Using OTOPLAN software and as shown in Figure 1, we determined the following cochlear parameters: A value, which represents the cochlear diameter by measuring the largest distance from the midpoint of the RW to the opposite cochlea lateral wall passing through the modiolus; B value, which resembles the cochlear base width in the form of a line passing through the mid-modiolus and perpendicular to the A value; and H value, which is the distance from the lowest point of the basal turn to the apex through the center of the modiolus. OTOPLAN software was used to calculate the CDL. 

### 2.3. Cochlear Impedance and Evoked Compound Action Potential (ECAP)

All patients underwent CI surgery with a standard mastoidectomy and posterior tympanotomy approach. After the insertion of the electrode array through the round window, implant telemetry was recorded using Maestro software (Med-El) (Version 10.0). Impedance telemetry from all 12 channels was measured using the manufacturer’s protocol, with amplitude and pulse widths of approximately 300 μA and 27 μs, respectively. Electrodes with high-impedance or short circuits that have been identified in the software were excluded. After impedance telemetry was measured, the intraoperative ECAP was recorded using Maestro software (Med-El) with a built-in automated ECAP measurement tool. We defined apical electrodes as (1–4), middle as (5–8), and basal as (9–12). All collected data were investigated for any correlation with CDL.

### 2.4. Statistical Analysis

After discussing the protocol and the objective of this study and conducting data collection and verification, all data were submitted for statistical analysis using R Software version 4.2.2 “Innocent and Trusting”. 

Descriptive analysis of patients’ demographics as well as baseline measures were conducted to determine quantitative data including mean, standard deviation, and range; for qualitative categorical variables, count and percentage were determined.

#### Analytical Statistics

- An investigation of the correlation between cochlear parameters and electrode impedance and electrically evoked compound action potential (ECAP) measures was conducted via Pearson correlation coefficients.
- Simple and multiple linear regression models were used to predict the amount of change in electrode impedance, and ECAP measures were determined based on every unit change in cochlear parameters.
- Normality assumptions were checked using the Shapiro–Wilk test.
- *p* values ≤ 0.05 were considered statistically significant.

## 3. Results

This study was carried out on 45 CI children: males represented about 57.8% and females represented 42.2%. The mean age at implantation was 2.4 ± 0.9 years. About 53.3% of the patients received left ear implants and the remaining 46.7% received right ear ones. Regarding electrode type, about 55.5% of the patients used Form 24, about 26.7% used Flex 28, and the remaining 17.8% used Flex 26. 

The mean values of the cochlear parameters were as follows: A value of 8.5 ± 0.4 mm; B value of 6.3 ± 0.4 mm; H value of 3.6 ± 0.4 mm; cochlear duct length of 33.2 ± 2.0 mm; and cochlear coverage of 76.0 ± 5.7%. 

The mean intraoperative cochlear impedance values were as follows: apical impedance of 5.5 ± 1.7 KΩ; middle impedance of 5.0 ± 1.6 KΩ; and basal impedance of 5.2 ± 1.4 KΩ. The overall average impedance was 5.2 ± 1.3 KΩ.

The mean intraoperative ECAP thresholds were as follows: apical ECAP of 13.1 ± 3.8; middle ECAP of 14.3 ± 3.7; and basal ECAP of 15.6 ± 4.8. The overall average ECAP was 14.3 ± 2.8. Patients’ demographics and baseline measures are presented in Table 1.

**Figure 2:**
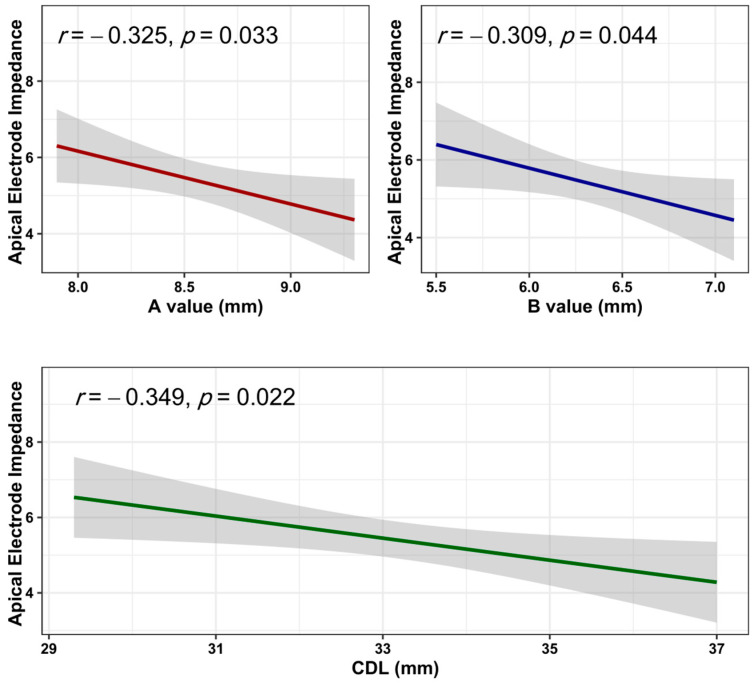
Correlation between cochlear parameters and significant electrode impedance measures.

The correlation between cochlear parameters (A value, B value, and CDL) and electrode impedance showed statistically significant weak relationships with apical impedance measures in negative directions (r = −0.325, −0.309, and −0.349, respectively) and *p* values = 0.033, 0.044, and 0.022, respectively. All correlations between cochlear parameters and electrode impedance are provided in Table 2, and those with significant relationships are shown in Figure 2.

**Figure 3:**
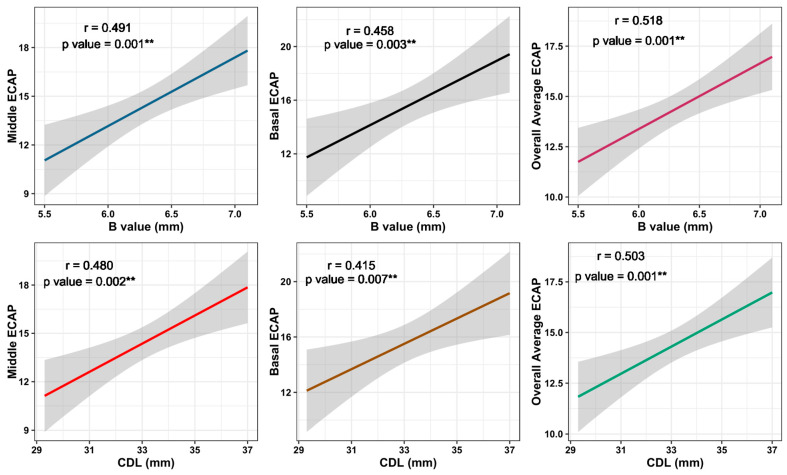
Correlation between cochlear parameters and significant ECAP measures. ** means statistically significant.

The correlation between the cochlear parameters and ECAP thresholds revealed that the B value and CDL showed statistically significant relationships with each of the middle ECAP thresholds (r = 0.491 and 0.480, *p* values = 0.001 and 0.002, respectively), with the basal ECAP thresholds (r = 0.458 and 0.415, *p* values = 0.003 and 0.007, respectively), and with the overall average ECAP thresholds (r = 0.518 and 0.503, *p* values = 0.001 for both, respectively). All correlations between the cochlear parameters and ECAP thresholds are presented in Table 2, and those with significant results are shown in Figure 3.

The percentage of cochlear coverage showed a statistically significant weak relationship in a negative direction with the apical ECAP thresholds (r = −0.395, *p* value = 0.011).

### 3.1. The Linear Regression Models between Electrode Impedance and Cochlear Parameters

**Figure 4:**
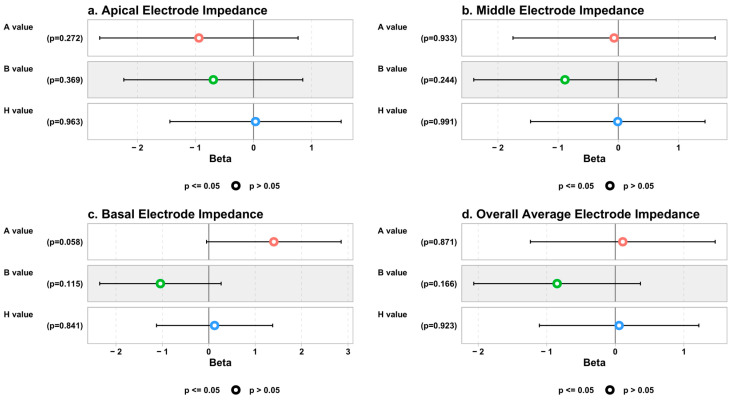
Association between cochlear parameters and electrode impedance measures (β coefficients and *p* values).

The univariate analysis for electrode impedance measures showed that for each one-unit increase in both the A and B values, the apical impedance values decreased significantly by about 1.38 and 1.22 units, respectively: β = −1.38, 95% CI (−2.66 to −0.11), *p* = 0.033, and β = −1.22, 95% CI (−2.40 to −0.04), *p* = 0.044. These lost their significance after adjusting for all the cochlear dimensions (A, B, and H values together) in the multivariate model. The details of the linear regression models between electrode impedance and cochlear parameters are shown in Figure 4.

### 3.2. The Linear Regression Models between the ECAP and Cochlear Parameters

Regarding the ECAP measures, the univariate analysis showed that for each one-unit increase in the B value, the middle ECAP thresholds increased significantly by about 4.23 and 4.27 units after adjusting for the A and H values in the multivariate analysis, respectively: univariate β = 4.23, 95% CI (1.83 to 6.62), *p* = 0.001, and multivariate β = 4.27, 95% CI (1.12 to 7.42), *p* = 0.009.

**Figure 5:**
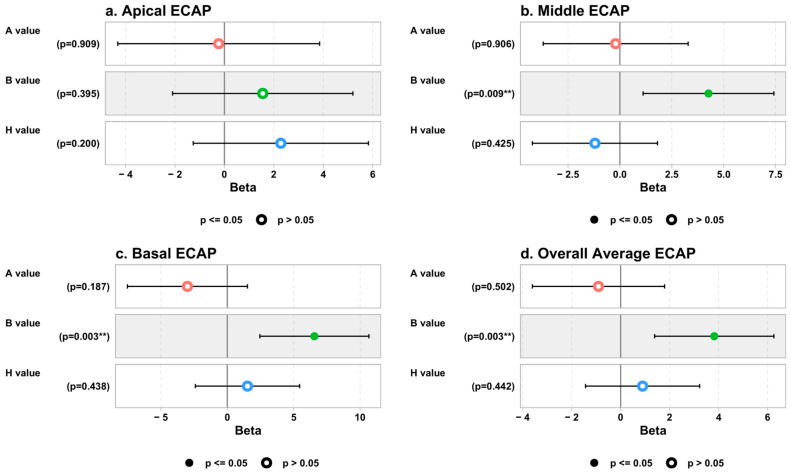
Association between cochlear parameters and ECAP thresholds (β coefficients and *p* values). ** refers to statistically significant.

The univariate analysis also showed that for each one-unit increase in B value, the basal ECAP thresholds increased significantly by about 4.81 and 6.56 units after adjusting for the A and H values in the multivariate analysis, respectively: univariate β = 4.81, 95% CI (1.64 to 7.98), *p* = 0.004, and multivariate β = 6.56, 95% CI (2.45 to 10.66), *p* = 0.003. Moreover, the univariate analysis showed that for each one-unit increase in B value, the overall average ECAP thresholds increased significantly by about 3.27 and 3.82 units after adjusting for the A and H values in the multivariate analysis, respectively: univariate β = 3.27, 95% CI (1.42 to 5.12), *p* = 0.001, and multivariate β = 3.82, 95% CI (1.39 to 6.26), *p* = 0.003. The details of the linear regression models between the ECAP and cochlear parameters are shown in Figure 5.

## 4. Discussion

Our results showed that the anatomical parameters of the cochlea, including the A, B, and H values, as well as the CDL, had a relationship with the ECAP thresholds in a positive direction in different cochlear regions. Interestingly, the B value showed a significant moderate correlation with the ECAP in the middle and basal electrodes but not in the apical electrodes. Additionally, linear regression models demonstrated that increasing the B value by one unit resulted in a significant increase in the ECAP thresholds in the middle, basal, and overall values. In this study, we found no significant correlation between the cochlear parameters and ECAP thresholds in apical regions, which could be due to our population anatomy or the cochlear coverage that was included. Söderqvist et al. investigated the ECAP thresholds in association with inner ear dimensions including cochlear diameter and bony cochlear nerve canal (BCNC). They reported higher ECAP thresholds in the larger cochlea and BCNC than smaller ones, even in different insertion angles, which could be explained by the increase in the cochlea diameter and the electrode–modiolar distance [16]. This finding is consistent with our results, as the anatomical parameters correlated with the ECAP thresholds in a positive direction. Notably, the B value, which identifies the cochlea width, showed a significant correlation on both middle and basal electrodes and thus could affect the position of the electrode and its distance to neurons. Moreover, another study exploring the variation in cochlear anatomy reported that the B value has a higher correlation with the CDL and cochlear volume than the A value [17]. This corroborated our findings about the B value rule and its impact on perioperative assessment during CI. Recognizing the anatomical variety in the cochlea can influence the location of the intracochlear electrode and postoperative speech results [18,19,20]. Our finding that the B value correlated with the ECAP thresholds could assist in electrode selection; however, further research is required to validate this correlation.

The cochlear duct volume expands as the scala tympani (ST) grows in height and width, and this leads to greater modiolar distance as the ST cross-sectional area becomes larger; this factor might contribute to the high ECAP thresholds in the larger cochlea [21,22,23,24]. We observed that the ECAP thresholds were smaller in the apical regions than the basal and thus determined that less electrical stimulation is required to induce the ECAP response in the apical electrodes, which is a finding similar to those of other studies [9,16]. A report by Lambriks et al. showed that the electrode in the apical region has greater ECAP responses than basal electrodes, and this could be explained by the greater number of SNGs observed toward the apex and the small diameter of the apex, which led to the apical electrodes being in close proximity to the modiolus neurons [10]. Another report by Zhu et al. showed that the physiological function of the peripheral auditory system is somewhat determined by the anatomy of the cochlea and that a better auditory conduction function is correlated with a larger cochlear size [25]. These findings were in line with our own, as the cochlear anatomy could affect CI surgery results and predict postoperative outcomes [25].

Electrode impedance indicates the degree of resistance to electrical current flow between stimulating and receiving electrodes, and the resistive characteristics and tissues surrounding the electrode can affect its values [26,27]. This study demonstrated that electrode impedance has a correlation in negative directions with different anatomical parameters and that there was a significantly weak relationship between the A and B values and the apical region. We observed higher impedances in apical electrodes compared with basal electrodes. In comparison, some studies reported similar findings to ours, as the impedances of the cochlear apex are greater than the basal area [10,26,27,28], while others found a reverse correlation [29]. Lambriks et al. found a weak to moderately significant association of electrode–modiolus distance and insertion depth with intraoperative impedances, as the electrodes with a short distance to the modiolus and toward the cochlear apex have higher impedances [10]. However, others have studied this association and found no clear relationship [28,29,30]. Although we did not address the electrode–modiolus distance in the present study, our findings showed that anatomical variation in cochlear parameters can affect electrode impedances, and this observation could assist in predicting CI outcomes. Our findings agreed with a study that reported that the cochlea size appears to be a key element that influences CI outcomes [31]. Another study showed that speech outcomes have a significant correlation with cochlear height in CI patients [32]. These anatomical factors should be incorporated into future electrode selection [31]. 

To the best of our knowledge, this is the first study to investigate the correlation between the A, B, and H values and intraoperative objective measures, including the ECAP thresholds and electrode impedance during CI, as an indirect measurement tool to assess SNG status and predict if these anatomical parameters influence cochlear objective measures intraoperatively. Hence, understanding the anatomical variation in the cochlea can affect the position of the intracochlear electrode, as well as postoperative speech outcomes [9]. Additionally, the consideration of anatomical variation could assist in preserving residual hearing and lead to a less traumatic approach to the cochlea. Moreover, the B value could have a considerable effect on the ECAP threshold in the middle and basal areas, which is highlighted in our results, making this observation important during perioperative workup for CI surgery. However, the present study has limitations, including its design as a retrospective study and the relatively small population that was included. Although we only focused on intraoperative measures, this study demonstrates findings that could shed light on future studies. Further studies with larger sample sizes are required to explore these measures and compare them with postoperative outcomes.

## 5. Conclusions

In summary, the results of our study demonstrate that intraoperative ECAP thresholds have a correlation with different cochlear parameters. The B value showed a significant association with the ECAP thresholds in the middle and basal electrodes. This variation could assist in optimizing the selection of the cochlear implant electrode array. Further research is required to study this correlation and CI outcomes postoperatively.

## Figures and Tables

**Table 1:** Descriptive analysis for patients’ demographics and baseline measures.

Demographics		All (N = 45)
Gender	Female	19 (42.2)
	Male	26 (57.8)
Age (Years)	Mean (SD)	6.6 (1.6)
	Min–Max	3.70–9.80
Ear	Left	24 (53.3)
	Right	21 (46.7)
Age at Implantation (Years)	Mean (SD)	2.4 (0.9)
	Min–Max	0.80–4.30
Electrode Type	Flex 26	8 (17.8)
	Flex 28	12 (26.7)
	Form 24	25 (55.5)
Cochlear Parameters		
A Value	Mean (SD)	8.5 (0.4)
	Min–Max	7.90–9.30
B Value	Mean (SD)	6.3 (0.4)
	Min–Max	5.50–7.10
H Value	Mean (SD)	3.6 (0.4)
	Min–Max	2.10–4.40
CDL	Mean (SD)	33.2 (2.0)
	Min–Max	29.30–37.00
Cochlear Coverage (%)	Mean (SD)	76.0 (5.7)
	Min–Max	61.00–89.00
Cochlear Impedances (kOhm)		
Apical	Mean (SD)	5.5 (1.7)
	Min–Max	2.90–9.67
Middle	Mean (SD)	5.0 (1.6)
	Min–Max	3.20–10.48
Basal	Mean (SD)	5.2 (1.4)
	Min–Max	3.34–8.41
All Impedances Average	Mean (SD)	5.2 (1.3)
	Min–Max	3.41–9.06
ECAP Thresholds (µV)		
Apical	Mean (SD)	13.1 (3.8)
	Min–Max	7.65–22.55
Middle	Mean (SD)	14.3 (3.7)
	Min–Max	8.20–21.78
Basal	Mean (SD)	15.6 (4.8)
	Min–Max	7.75–28.15
All ECAPs Average	Mean (SD)	14.3 (2.8)
	Min–Max	8.98–22.76

Data are represented as mean (standard deviation) and frequency (percentage).

**Table 2:** Correlation between cochlear parameters and cochlear impedances and ECAP thresholds.

		Apical	Middle	Basal	Overall Average
Regarding Cochlear Impedance Measures kOhm					
A Value	Coefficient (r) *p* Value	−0.325 (0.033)	−0.160 (0.305)	0.209 (0.179)	−0.132 (0.398)
B Value	Coefficient (r) *p* Value	−0.309 (0.044)	−0.248 (0.109)	−0.083 (0.597)	−0.263 (0.088)
H Value	Coefficient (r) *p* Value	−0.029 (0.852)	0.011 (0.944)	0.141 (0.365)	0.043 (0.783)
CDL	Coefficient (r) *p* Value	−0.349 (0.022)	−0.262 (0.090)	−0.015 (0.925)	−0.263 (0.089)
Cochlear Coverage (%)	Coefficient (r) *p* Value	−0.036 (0.817)	−0.175 (0.261)	−0.084 (0.592)	−0.122 (0.436)
**Regarding Compound Action Potential (ECAP) Measures µV**					
A Value	Coefficient (r) *p* Value	0.137 (0.394)	0.268 (0.090)	0.162 (0.312)	0.281 (0.075)
B Value	Coefficient (r) *p* Value	0.162 (0.313)	0.491 (0.001)	0.458 (0.003)	0.518 (0.001)
H Value	Coefficient (r) *p* Value	0.190 (0.234)	−0.173 (0.279)	−0.007 (0.964)	0.008 (0.961)
CDL	Coefficient (r) *p* Value	0.177 (0.268)	0.480 (0.002)	0.415 (0.007)	0.503 (0.001)
Cochlear Coverage (%)	Coefficient (r)*p* Value	−0.395 (0.011)	−0.027 (0.865)	−0.107 (0.507)	−0.243 (0.125)

Computed correlation used Pearson method with listwise deletion.

## Data Availability

The datasets generated during and/or analyzed during the current study are available from the corresponding author upon reasonable request.
